# The dynamic changes in the number of uterine natural killer cells are specific to the eutopic but not to the ectopic endometrium in women and in a baboon model of endometriosis

**DOI:** 10.1186/s12958-018-0385-3

**Published:** 2018-07-18

**Authors:** Josephine A. Drury, Kirstin L. Parkin, Lucy Coyne, Emma Giuliani, Asgerally T. Fazleabas, Dharani K. Hapangama

**Affiliations:** 1 Department of Women’s and Children’s Health, Institute of Translational Medicine, University of Liverpool, Liverpool, UK; 2 Department of Obstetrics, Gynecology and Reproductive Biology, College of Human Medicine, Michigan State University, Grand Rapids, MI USA; 3 Department of Microbiology and Molecular Genetics, Michigan State University, East Lansing, MI USA; 4 Department of Gynecology, Liverpool Women’s Hospital, Liverpool, UK; 5 Hewitt Fertility Centre; Liverpool Women’s Hospital, Liverpool, UK; 6 Department of Obstetrics and Gynecology, Grand Rapids Medical Education Partners/Michigan State University, Grand Rapids, MI USA

**Keywords:** Uterine natural killer cells, Endometriosis, Humans, Primate, Baboon

## Abstract

**Background:**

Endometriosis is a common condition associated with growth of endometrial-like tissue beyond the uterine cavity. Previous reports have suggested a role for uNK cells in the pathogenesis of endometriosis postulating that survival and accumulation of menstrual endometrial tissue in the peritoneal cavity may relate to a reduction in the cytotoxic activity of peripheral blood NK cells. We aimed to assess the differences in percentage of uNK cells and their phenotypical characterization in eutopic and ectopic endometrial samples from women with and without endometriosis and baboons with induced endometriosis.

**Methods:**

Eutopic and ectopic endometrial samples from 82 women across the menstrual cycle with/without endometriosis and from 8 baboons before and after induction of endometriosis were examined for CD56 and NKp30 expression with immunohistochemistry, quantified using computer assisted image analysis. Curated secretory phase endometrial microarray datasets were interrogated for NK cell receptors and their ligands. In silico data was validated by examining the secretory phase eutopic endometrium of women with and without endometriosis (*n* = 8/group) for the immuno-expression of BAG6 protein.

**Results:**

The percentage of uNK cells increased progressively from the proliferative phase with the highest levels in the late secretory phase in the eutopic endometrium of women with and without endometriosis. The percentage of uNK cells in ectopic lesions remained significantly low throughout the cycle. In baboons, induction of endometriosis increased the percentage of uNK in the ectopic lesions but not NKp30. Published eutopic endometrial microarray datasets demonstrated significant upregulation of NKp30 and its ligand BAG6 in women with endometriosis compared with controls. Immunohistochemical staining scores for BAG6 was also significantly higher in secretory phase eutopic endometrium from women with endometriosis compared with the endometrium of healthy women (*n* = 8/group).

**Conclusions:**

The dynamic increase in the percentage of uNK cells in the secretory phase is preserved in the endometrium of women with endometriosis. The low number of uNK cells in human and baboon ectopic lesions may be due to their exaggerated reduction in hormonal responsiveness (progesterone resistance).

**Electronic supplementary material:**

The online version of this article (10.1186/s12958-018-0385-3) contains supplementary material, which is available to authorized users.

## Background

[1, 2] Endometrial leucocytes are postulated to play an important role in normal endometrial functions [3] and CD56^bright^ CD16^−^ uterine Natural killer (uNK) cells are the predominant leucocyte subset in the secretory phase endometrium [4]. They are likely to have functions in inflammatory modulation, angiogenesis, apoptosis, and extracellular matrix remodelling and these activities may continue into the decidual tissue of the very early stages of pregnancy [5, 6]. NK cells are terminally activated by specific receptors such as NK cell p30 related protein (NKp30) receptor, through their corresponding ligands which are up-regulated on the surface of cells that are deemed to be a threat to the body, such as cancer cells [7]. Intriguingly, decidual uNK cells, which have attenuated cytotoxicity [8] express NKp30, and most available data on uNK cells focus on pregnant decidua while the evidence regarding NKp30 expression in non-pregnant uNK cells is limited.

The purported importance of uNK cells, and in particular their numbers, is well documented in the pathogenesis of a variety of female reproductive disorders such as recurrent miscarriage [9], sporadic miscarriage [10], recurrent implantation failure [11], fibroids [12], fetal growth restriction and pre-eclampsia [13].

Endometriosis is a common, benign, chronic inflammatory gynaecological disease often associated with subfertility [14], characterized by the presence of endometrial glands and stroma-like tissue outside the uterine cavity [14]. The eutopic endometrium of women with endometriosis has been shown to be different to that of women without endometriosis [14–17] while persistent proliferation and progesterone resistance is known to exist in ectopic lesions [14, 16, 18, 19]. The pathogenesis of endometriosis is not fully understood, although the theory of retrograde menstruation, where subsequent deposition of shed endometrium in the pelvic cavity gives rise to endometriotic deposits, is the most widely accepted [14]. Previous reports have suggested a role for uNK cells in the pathogenesis of endometriosis [20–22] postulating that survival and accumulation of menstrual endometrial tissue in the peritoneal cavity may relate to a reduction in the cytotoxic activity of peripheral blood NK cells [23]. Jones et al. investigated various leukocyte subpopulations in endometriosis and adenomyosis, however, the data is expressed relative to the number of leukocyte antigen positive cells [21]. There are no comprehensive studies that describe the uNK cell numbers relative to the endometrial stromal niche cells in eutopic and ectopic endometrium of women with endometriosis published to date that utilise a validated analytic method to ensure reproducibility or generalisability of data. Furthermore, cycle phase specific changes in uNK cell numbers including proliferative phase, mid-secretory and late-secretory phase in relation to endometriosis have not yet been described. Studying the establishment of the disease in humans is challenging since it is impossible to know how long the disease has been present at the point of surgical diagnosis and the correlation between symptoms and disease severity is poor. The baboon model of induction of endometriosis thus provides a unique opportunity to study the natural course of endometriosis following the initial establishment of the disease [24].

Since uNK cells are of great interest to reproductive biologists and immunologists as a target for therapies, we aimed to assess the uNK cell numbers and their NKp30 activation status in a well characterised patient population with or without endometriosis across different phases of the menstrual cycle and to examine the early stages of disease establishment in the baboon model of induction of endometriosis. Ectopic lesions excised from women and baboons were also examined and compared to the eutopic endometrium. To overcome the deficiencies in previous publications on the subject we employed a validated and reproducible computer assisted tool [25] in our analysis and further examined curated micro-array data, which was validated by examining the differential expression of one of the identified gene products (BAG6) in the eutopic endometrium of women with and without endometriosis.

## Methods

Endometrial biopsies were taken from 30 patients with surgically diagnosed peritoneal endometriosis at American Fertility Society stages I–IV and 30 healthy fertile controls (at least one live birth without a history of subfertility, recurrent miscarriage or endometriosis, confirmed by laparoscopy) undergoing laparoscopic sterilization [16] at Liverpool Women’s Hospital, Liverpool, UK (tertiary referral centre). All women included had regular menstrual cycles (26–30 days), were not on any hormonal therapy and were not using an intrauterine device. Endometrial biopsies were grouped by cycle stage: 10 proliferative, 10 mid-secretory and 10 late-secretory phase per group, with cycle stage confirmed by histological dating according to modifications of Noyes criteria [26]. Samples were fixed in 10% buffered formalin for 24 h prior to embedding in paraffin blocks for immunohistochemistry.

### Ectopic lesions: Human

Peritoneal red/blue ectopic lesions (no ovarian or deep infiltrating endometriosis lesions were included) histologically confirmed to contain endometrium-like cells (glandular and or stromal components) were excised from 22 patients (day 2 to day 30 of menstrual cycle; 2 menstrual, 6 proliferative, 10 mid-secretory, 4 late secretory). Seven of these also had matched eutopic endometrial biopsies.

### Baboon samples

Tissues obtained from previously well-described baboon model of endometriosis induction was utilised for this study [24, 27–29]. As previously described [24], animals were housed in the animal care facility at the University of Illinois, Chicago, USA, and all studies were approved by the University of Illinois IACUC. Laparoscopy confirmed the absence of spontaneous endometriosis and endometrium was harvested from each animal at day 9 to 12 post-ovulation, prior to the induction of endometriosis (control, *n* = 5). Endometriosis was then induced in ten female baboons (*Papio anubis)* by intra-peritoneal inoculation of autologous menstrual endometrial tissue on the first or second day of menstruation on two consecutive menstrual cycles, as previously reported [24]. Disease progression was monitored by consecutive laparoscopies and video recording at 3 (*n* = 8), and 15 months (n = 8) after induction of endometriosis. Following each laparoscopy, a laparotomy was performed and eutopic/ectopic endometrial tissue was harvested at day 9–12 post-ovulation. The animals were euthanized at 15 months post-induction as required by the IACUC approval.

### Ectopic lesions: Baboon

Blue ectopic lesions were harvested at day 9–12 post-ovulation at 3 months (*n* = 4) and 15 months (*n* = 5) post-inoculation. Each lesion was taken from a different animal.

### Immunohistochemistry

Expression of CD56, NKp30 and BAG6 was determined by immunohistochemistry. 3 μm (human) or 5 μm (baboon)-thick paraffin sections were incubated with either monoclonal mouse anti-human CD56 (NCAM, clone 1B6 Novocastra Leica Biosystem, Newcastle, UK) antibody at 1:50, polyclonal goat anti-human NKp30 antibody (sc-20,477, Santa Cruz Biotechnology, Inc) at 1:100 dilution or polyclonal rabbit anti-human BAG6 antibody (HPA053291, ATLAS antibodies, Cambridge Biosciences UK) at 1:500 for 1 h at room temperature in a humidified chamber. Detection was with ImmPRESS anti-mouse, anti-goat or anti-rabbit polymer (Vector Laboratories, Peterborough, UK) respectively and visualisation was with ImmPACT DAB (Vector Laboratories, Peterborough, UK). The sections were counterstained in Gill 2 Haematoxylin, dehydrated, cleared and mounted in Consul Mount (Thermo Scientific, Runcorn, UK). Mouse, goat or rabbit negative control IgG (0.5 μg/ml Vector Laboratories, Peterborough, UK) replaced the respective primary antibody as a negative control.

### Image analysis

Ten high-resolution images were captured using a Nikon Eclipse 50i Microscope, Nikon Corporation, Surrey, UK and Nikon DS Fi1 digital camera (Nikon) at 400× magnification for each sample and edited to leave only stromal cells. The ratio of the area occupied between positive CD56 or NKp30 cells (brown stain) and total endometrial stromal cells (blue stain) was assessed using computer assisted image analysis with color deconvolution (Image J software, NIH) for each image (10 images for each sample) [25]. The average percent of positive staining as a total of the stromal cells present was then calculated for each sample (previously shown to be equivalent to counting uNK cells) [25]. The investigators were blinded to the identification of the endometrial tissue sections during the analysis.

#### Semi-quantitative quickscore for BAG6

The immunostaining was first broadly evaluated to identify the location of the positively stained areas. Subsequently, the *functionalis* glands from each section were analysed semi-quantitatively using a modified Quickscore method incorporating both staining intensity and abundance [30–32].

### Bioinformatics analysis

The role of key receptors on human NK cells was examined by collating a list of inhibitory and activating receptors, adhesion molecules or co-stimulatory molecules [33]. Curated datasets containing microarray data from secretory phase patients with endometriosis (*n* = 60; 24 early-secretory and 36 mid-secretory phase) compared with normal endometrium (*n* = 25; 9 early-secretory and 16 mid-secretory phase) [34, 35] were examined using the meta-analysis function in the Illumina BaseSpace Correlation Engine for the gene list described above and tabulated.

### Statistical analysis

Graphpad prism was used for all analyses. Cell densities of related and non-related groups were compared by non-parametric tests as appropriate (Kruskall Wallis and Mann–Whitney *U*-test). Parametric and non-parametric tests were used to compare differences between groups as appropriate. Data are presented as median (range). Statistical significance was set at *P* < 0.05.

## Results

### Demographic characteristics

There were no statistically significant differences in age, BMI or smoking status between the two groups of women although the control group tended to be older (Table 1).

**Table 1:** Clinical characteristics of study women

Demographic data	Control group *N* = 30	Endometriosis group *N* = 30	Ectopic group *N* = 22	*P* value (control v endometriosis)
Age, median (range)	40 (25–47)	36 (18–45)	40 (24–51)	*P* = 0.056
BMI, median (range)	27 (20–42)	26 (20–38)	27.1 (18–32)	*P* = 0.37
Parity, median (range)	2 (1–4)	1 (0–3)	1 (0–2)	P < 0.0001
Smoker	9/30 (30%)	5/30 (17%)	1/22 (5%)	*P* = 0.36
Endometriosis stage, median (range)	–	2 (1–4)	4 (1–4)	N/A

Control group consists of 10 patients with proliferative phase endometrium, 10 patients with mid-secretory phase endometrium and 10 patients with late-secretory phase endometrium. Endometriosis group consists of 10 patients with proliferative phase endometrium, 10 patients with mid-secretory phase endometrium and 10 patients with late-secretory phase endometrium. The ectopic group consists of ectopic lesions excised from women with endometriosis, 2 menstrual, 6 proliferative, 10 mid-secretory and 4 late secretory phase. Mann-Whitney U test for age, BMI and parity; Fisher’s Exact test for smoking status

Parity was significantly higher in the control group (*P* < 0.0001). However, this was expected since proven fertility was part of the inclusion criteria for this cohort of women.

In the baboons, the average number of endometriotic lesions after the inoculation of endometrial tissue was 20.2 ± 11.5 at 3 months and 20.3 ± 8.1 at 15 months. No lesions were visualized before the induction of the disease in any animal.

### The dynamic CD56 and NKp30 expression pattern observed in human eutopic endometrium across the cycle is preserved in women with endometriosis

**Fig. 1:**
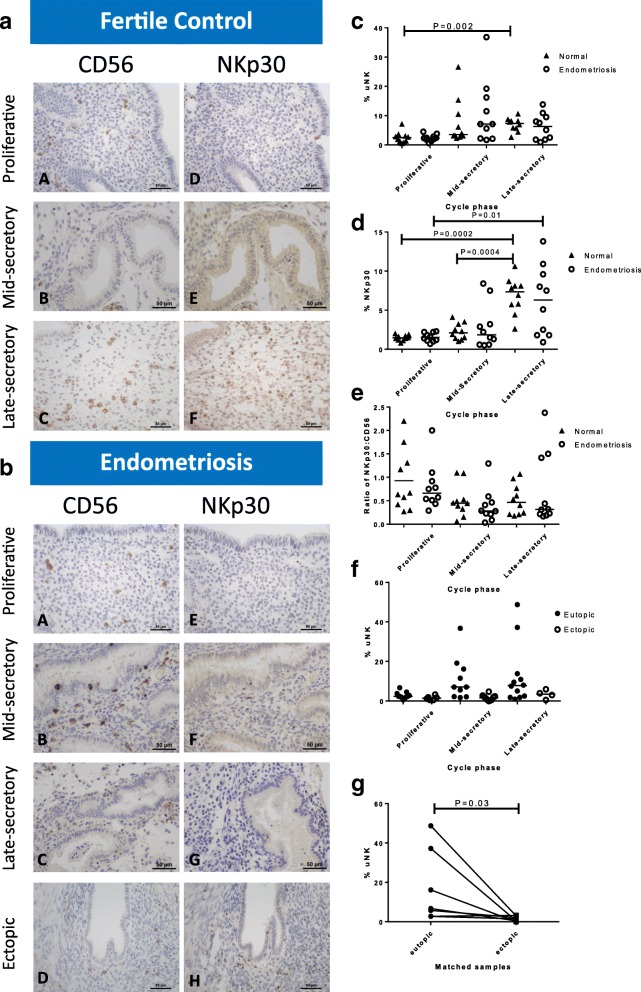
Expression of CD56 and NKp30 in human endometrium. Representative micrographs showing CD56 expression by immunohistochemistry (brown DAB staining) in eutopic endometrial stromal cells of fertile control women (**a**, *A*-*C*) and in women with endometriosis (**b**, *A*-*C*) (400× magnification). NKp30 expression in eutopic endometrial stromal cells from fertile control women (**a**, *D*-*F*) and women with endometriosis (**b**, *E*-*G*). Staining in ectopic lesions are shown in (**b** *D*) (uNK cells) and (**b** *H*) (NKp30). Graphs comparing %CD56 (1c), %NKp30 (**d**) and ratio of NKp30:CD56 (**e**) in ectopic lesions (*n* = 6–9) and at different time points in the menstrual cycle (PP = proliferative phase; MSP = mid-secretory phase; LSP = late-secretory phase; *n* = 10 for each group in human samples (in both fertile controls ‘normal’ and patients with endometriosis). (**f**) Graph showing percentage of CD56+ uNK cells in eutopic endometrium and ectopic lesions across the menstrual cycle (*n* = 9 ectopic lesions with matched eutopic endometrium in 7/36 cases) demonstrating that levels remain low in ectopic lesions. (**g**) Graph showing percentage of CD56+ uNK cells in matched eutopic and ectopic endometrium (*n* = 7). *P* = 0.03, Wilcoxon matched pairs signed rank test

**Fig. 2:**
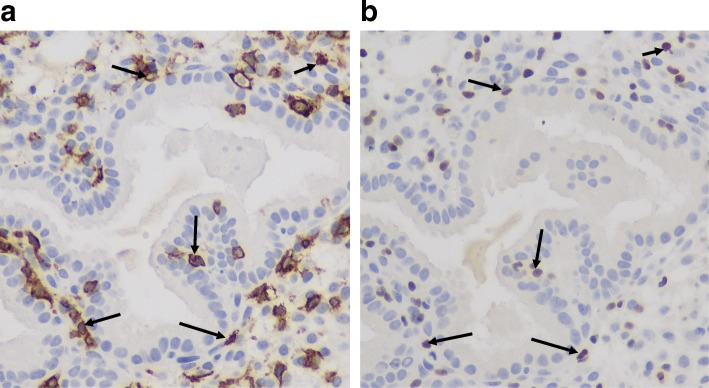
Co-localisation of CD56 (**a**) and NKp30 (**b**) positive cells on serial sections from late secretory endometrium. Examples of cells stained with both markers are shown by black arrows

In fertile control women, the percentage of uNK cells in the stromal compartment rose significantly in the eutopic endometrium across the menstrual cycle (Kruskal-Wallis test *P* = 0.0038), with the highest levels seen in the late secretory phase (7.35% (2.6–10.6)). Mann Whitney U test showed significantly higher CD56 in eutopic endometrial biopsies taken from fertile control women in the late secretory phase compared with proliferative phase (*P* = 0.002, Fig. 1c). Although the same trend was seen across the menstrual cycle in the eutopic endometria of women with endometriosis, the increase bordered on statistical significance (Kruskal-Wallis test *P* = 0.05). However it was noted that there appeared to be an earlier rise in the percentage of uNK cells in the endometriosis group - in the mid-secretory phase of the cycle (7.1% (1.7–36.8)) compared to the fertile control group (3.6% (2.3–26.6)). Eutopic endometrial CD56 co-localised with NKp30 on serial sections of late secretory endometrium (Fig. 2). There was a statistically significant increase in %NKp30 across the cycle from proliferative to late-secretory phase eutopic endometrium in both the fertile control group and endometriosis group (Kruskal-Wallis test *P* < 0.0001 and *P* = 0.03 respectively, Fig. 1d). NKp30 was significantly higher in the late-secretory phase eutopic endometrium compared with proliferative phase in both fertile control (Mann Whitney U test *P* = 0.0002) and endometriosis patients (Mann Whitney U test *P* = 0.01). In fertile control patients, there was also a significant increase in NKp30 from mid-late secretory phase endometrium (Mann Whitney U test *P* = 0.0004). There was a strong correlation between CD56 and NKp30 in control patients (*r* = 0.63, P = 0.0002), whilst the correlation in the eutopic endometrium of endometriosis patients was not statistically significant (*r* = 0.35, *P* = 0.06) (Additional file 1: Figure S1). The ratio of eutopic endometrium NKp30:CD56 was calculated across the cycle to give an indication of relative uNK cell activation and had a small decrease across the menstrual cycle in the endometriosis group (Fig. 1e, Kruskal-Wallis test *P* = 0.05).

### CD56 expression in human ectopic endometriotic lesions

Ectopic lesions excised from women showed a low %CD56+ cells throughout the menstrual cycle (KW test *P* = 0.3), similar to the levels seen in proliferative phase eutopic endometrium (Fig. 1f). In the mid-secretory phase, %CD56+ was significantly lower in ectopic lesions than in eutopic endometrium (Mann Whitney U test *P* = 0.004, *n* = 10 per group). In paired eutopic and ectopic endometrium the percentage of uNK cells was significantly lower in the matched ectopic endometrium (*P* = 0.03, *n* = 7, Wilcoxon matched pairs signed rank test, Fig. 1g).

### CD56 and NKp30 expression in baboon eutopic endometrium with induction of endometriosis

**Fig. 3:**
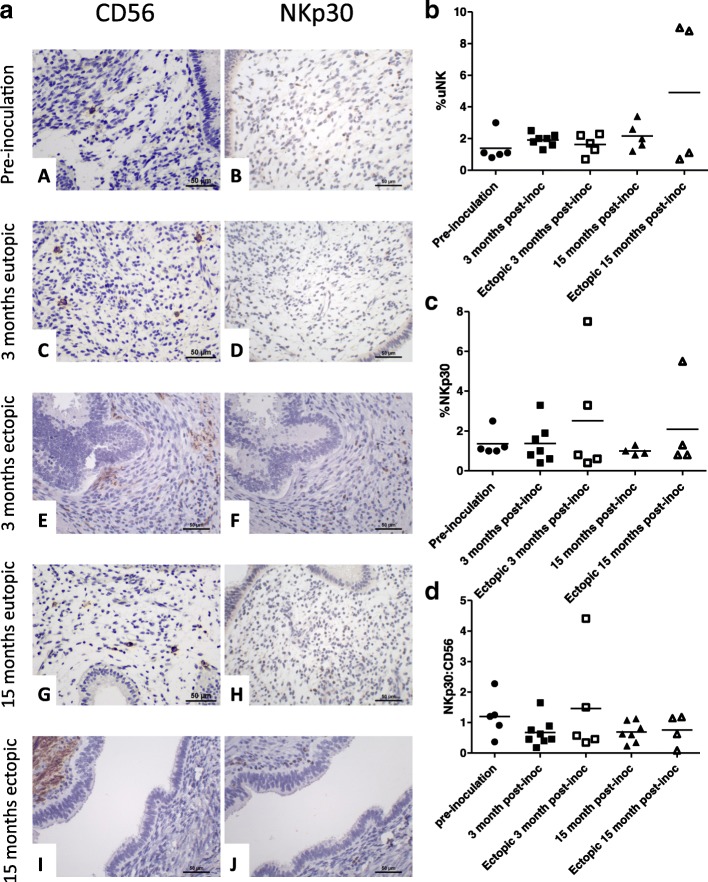
Expression of CD56 and NKp30 in a baboon model of induced endometriosis. **a** Representative micrographs depicting CD56 (*A, C, E, G, I*), or NKp30 (*B, D, F, H, J*) expression in eutopic endometrial stroma cells of baboon samples during the three time-points: pre-inoculation (*A, B*), 3 (*C, D*) and 15 months (*G, H*) post-inoculation of the disease and expression in ectopic endometrial lesions 3 months (*E, F*) and 15 months (*I, J*) post-inoculation (400× magnification). Graphs comparing percentage of stromal CD56^+^ (**b**), NKp30^+^ (**c**) and ratio of NKp30^+^ to CD56 (**d**) cells prior to inoculation (*n* = 5), at 3 (n = 7) and 15 months (n = 5) in eutopic endometrium after the induction of endometriosis and in ectopic lesions at 3 (n = 5) and 15 months (*n* = 4) after induction of endometriosis

Compared to pre-induction controls the median (range) %CD56^+^ cells (1.1% (0.8–3.0) *n* = 5; KW test, *P* = 0.17, Fig. 3) was not statistically significantly different at 3 months (2.0% (1.3–2.5) *n* = 8) and 15 months (1.8% (0.8–3.4) n = 8) in the eutopic endometrium post-induction of endometriosis although the median levels were slightly higher. The median %NKp30^+^ cells also remained similar after induction of endometriosis in the eutopic tissue. Interestingly, induction of endometriosis resulted in a trend to slightly lower ratio of NKp30:CD56 in the eutopic endometrium at 3 and 15 months compared to pre-induction controls (KW test *P* = 0.19, Fig. 3).

### CD56 and NKp30 expression in baboon ectopic endometriotic lesions

In baboons at 3 months post-induction of endometriosis, %CD56+ were similar in both eutopic endometrium and ectopic lesions (Fig. 3b). Yet, 15 months after the induction of endometriosis, the ectopic lesions from half of the animals (2/4) demonstrated three fold greater %CD56+ cells when compared with their eutopic endometrium (Fig. 3b). Furthermore, the %NKp30+ uNK cells at 3 months and 15 months post-induction of endometriosis appeared raised in the ectopic lesions from 1 or 2 animals when compared with eutopic endometrium (Fig. 3c).

### Bioinformatics analysis of differential expression of genes encoding NK cell receptors and ligands in secretory phase endometrium

Of the 92 genes examined, 60 were significantly up- or down- regulated (Additional file 2: Table S1) in endometrial samples of women with endometriosis relative to control women. The 10 most significantly up/down-regulated genes are shown below in Table 2.

**Table 2:** Top differentially regulated NK cell related genes in endometriosis

Gene	Gene Description	Specificity	Overall Gene score	Up/down-regulated
NCR3	natural cytotoxicity triggering receptor 3	6 out of 6	135.1	Up
SIGLEC7	sialic acid binding Ig-like lectin 7	5 out of 6	99.5	Up
CADM1	cell adhesion molecule 1	4 out of 6	234.7	Down
SELPLG	selectin P ligand	4 out of 6	158. 6	Up
COL1A1	collagen, type I, alpha 1	4 out of 6	158.1	Up
KIR3DL2	killer cell immunoglobulin-like receptor, three domains, long cytoplasmic tail, 2	4 out of 6	138.48	Up
BAG6	BCL2-associated athanogene 6	4 out of 6	136.0	Up
COL6A1	collagen, type VI, alpha 1	3 out of 6	162.0	Up
HCST	hematopoietic cell signal transducer	3 out of 6	154.9	Up
KIR2DS2	killer cell immunoglobulin-like receptor, two domains, short cytoplasmic tail, 2	3 out of 6	147.4	Up

Table showing the most significantly differentially regulated NK cell related genes examined. Specificity refers to the number of biological datasets in which the gene was found to be differentially regulated. Overall gene score is a measure of the fold change in the individual datasets combined with the number of datasets in which the particular gene was differentially regulated

NKp30 (NCR3) and its ligand BAT3 (BAG6) were significantly upregulated in 6/6 and 4/6 secretory phase datasets from endometriosis patients respectively compared with control patients (1.2–1.7 fold change and 1.4–2.3 fold change respectively, Additional file 2: Table S1).

In accordance with the immunohistochemistry data, NCAM1 (CD56) was not differentially regulated in 5/6 datasets.

Considering the other NK cell regulatory genes that were significantly altered in the majority of the endometriosis datasets (4–6/6), either a particular receptor (e.g. KIR3DL2) or the ligand (e.g. NECL2) was differentially expressed, but paired alteration of both the receptor and its ligand was not observed (Additional file 2: Table S1).

### In vivo validation of in silico data

**Fig. 4:**
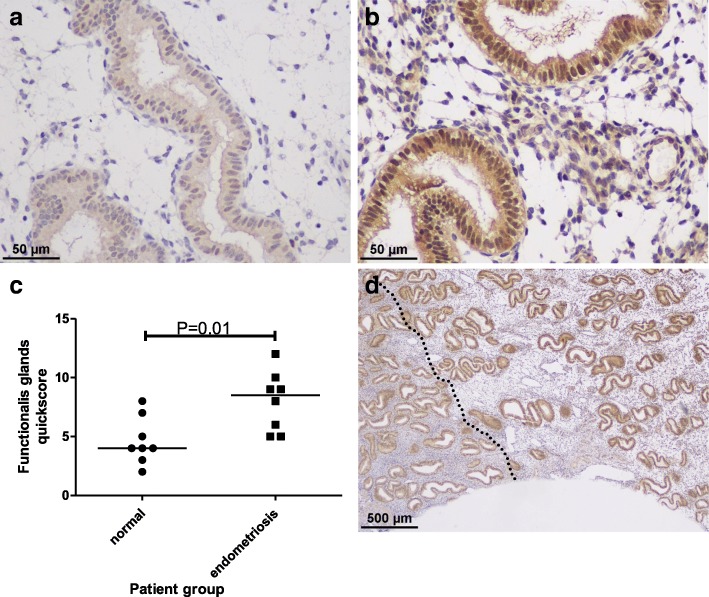
BAG6 expression in mid-secretory phase human endometrium. Representative micrographs depicting BAG6 expression in the functional layer of the endometrium from **a** fertile control women (400× magnification) and **b** women with endometriosis (400× magnification). **c** Quickscore data comparing BAG6 immuno staining in the endometrium of normal control women compared with women with endometriosis during the mid-secretory phase and demonstrating significantly increased BAG6 immunoexpression scores in the endometriosis group (*n* = 8/group, *P* = 0.01, Mann Whitney U test). In full thickness endometrium, a gradient in staining intensity was observed from the *functionalis* to the *basalis* layer (D, 40× magnification). The *basalis/functionalis* demarcation is indicated by the dotted line with the *basalis* to the left of the line and the *functionalis* to the right

We subsequently chose one of the gene products identified in our bioinformatics analysis, BAG6 for further study. BAG6 expression was not previously reported in the human endometrium, and we confirmed the expression of BAG6 protein in the endometrium. The strongest immuno-staining for BAG6 was in the endometrial epithelial compartment (highest quickscores in the luminal epithelium) but staining was also observed in stromal and vascular cells. Eutopic endometrium from women with endometriosis in the secretory phase showed significantly higher immunoexpression scores for BAG6 (Fig. 4) supporting our in silico data.

## Discussion

We have shown that the cyclical percentage change of uNK cells that occurs in healthy fertile endometrium, with a clear increase in the late-secretory phase of the cycle, is preserved in the eutopic endometrium of women with endometriosis. This observation was supported in the baboon model where induction of endometriosis was not associated with a significant increase in %CD56+ cells in the mid-secretory eutopic endometrial samples compared with pre-inoculation control samples. The use of the primate model of endometriosis (proposed to be the gold standard animal model of endometriosis) allowed us to document the precisely timed changes in eutopic uNK cells induced by the establishment of endometriosis, particularly at the very early stages of the disease, which is not feasible to attain in women due to the significant delay in diagnosis and poor correlation between symptoms and disease severity.

It is tempting to speculate that the animals with higher %uNK in ectopic lesions 15 months post-inoculation may be less likely to have lesions that persist as active endometriotic deposits and that those with low %uNK are able to evade the body’s immune surveillance mechanisms thus contributing to disease establishment. However, at present, there is insufficient evidence to suggest that uNK cells play a role in the establishment of ectopic endometriotic lesions despite the increasing evidence for a role in infertility [20, 36].

We have also demonstrated, that NKp30, an activating receptor of uNK cells, is expressed in endometrial uNK cells in the non-pregnant endometrium of humans and in baboons and that the NKp30 expression increases in the late secretory phase in humans. Furthermore, this increase of eutopic endometrial NKp30 expression and the highest level of NKp30 were observed in the late secretory phase of the cycle in women with/without endometriosis in agreement with some of the previous work [37]. Previous reports on NKp30 expression in uNK cells from non-pregnant endometrium are contradictory. FACS analysis of uNK cells isolated from mid-secretory phase did not show significant NKp30 expression [38] yet menstrual blood NK cells (with uNK phenotype, CD56^bright^, CD16^dim^) showed NKp30 expression [39]. Our data suggest a possible explanation to these seemingly contradictory reports. We propose that the menstrual blood NK cells studied by van der Molen et al. are likely to originate from the late-secretory endometrium. It seems that, in the late secretory phase, there is also an influx of other NKp30 expressing cells such as T cells [40–42]. Interestingly there was a strong, significant correlation between CD56+ cells and NKp30 in the normal eutopic endometrium which was lost in the endometriosis samples, further suggesting the NKp30+ expressing cells in women with endometriosis may be related to a T cell subpopulation.

In agreement with previous reports [21, 43] ectopic lesions had significantly low uNK cell numbers. Previous authors have also suggested that the ectopic lesions may have an increased number of CD3 and CD8 expressing T cells [21], which may express Nkp30 [42]. We also observed low Nkp30 expression in ectopic lesions, similar to the eutopic endometrium at the proliferative phase of the cycle. NKp30 is a natural cytotoxicity receptor (NCR) [38, 44], and ligand induced down-regulation of the receptor expression has been proposed as an immune surveillance evading mechanism in some tumors [45]. If low NKp30 expression is associated with reduced uNK cell cytotoxic activity, this may allow established human ectopic endometriotic cells to persist and evade immuno-clearance. Additional studies using the baboon model may help to determine whether NKp30 in uNK cells plays a role in propagation of ectopic lesions. Ectopic endometriotic lesions are postulated to have a progesterone resistant phenotype [46], which may further explain the observed low percentage of uNK cells in the human lesions.

The immune cell composition in the endometrium at the time of implantation is considered pivotal for successful conception; whereas at the end of the implantation window, during the late secretory phase, the main function of the endometrium is effective shedding and regeneration when no pregnancy ensues. Therefore, it is possible that as part of the innate immune system, uNK cells could play a role in both these contrasting functions of the eutopic endometrium as the most abundant leukocyte subpopulation in the human endometrium at both the mid and late secretory phase [47]. Endometriosis is a clinically challenging condition, associated with subfertility, with reported aberrations in mid-secretory phase endometrial function; whereas abnormal shedding of an aberrant late secretory phase endometrium [15, 19, 48] is postulated to explain its pathogenesis. Our data suggest that possible higher amounts of activated (NKp30 expressing) uNK cells in the eutopic endometrium of a subset of women with endometriosis may indicate possible functional aberrations in these cells in the late secretory endometrium. Further studies are warranted in the future to examine the functional differences in the production of cytokines and other immune modulators to determine how that may change the endometrial phenotype of the shedding endometrium of women with endometriosis.

Through systems biology and reviewing published literature, we have highlighted the complex nature of uNK cell activation and function. The final activation status or function of uNK cells will depend on the homeostasis of all the uNK cell activation/inhibitory receptors or the availability of the corresponding ligands, the vast majority of which were differentially regulated in the endometria of women with endometriosis. BAG6 is one of the ligands for NKp30, and was one of the genes identified in our in silico study (Table 2 and Additional file 1: Figure S1). BAG6 has multiple functions including apoptosis, gene regulation, protein synthesis, protein quality control, and protein degradation. We have demonstrated that human endometrium expresses BAG6 protein for the first time, and revealed an increased immuno-expression for BAG6 in secretory endometrium of women with endometriosis validating our in silico study. BAG6 has also been shown to be expressed on dendritic cells and cells after malignant transformation, where it serves as the ligand for NKp30 triggering NK cell cytotoxicity [49]. Further studies are warranted to elucidate the exact functional relevance of the presence of this protein in the endometrium.

Furthermore, we have previously published evidence for eutopic endometrial gene expression alterations subsequent to the induction of ectopic endometriotic lesions in baboons [29]. These published changes in eutopic endometrial gene expression included many of the endometriosis specific eutopic endometrial gene alterations reported in the human [29]. Interestingly, 40 of the 92 genes encoding NK cell receptors and ligands in our list were also amongst the differentially regulated gene list in the baboon eutopic endometrium at 6 months after induction of endometriosis (reported in Additional file 3: Table S2 in Afshar et al. [29], and in Additional file 3: Table S2), suggesting a close homology between the baboon model of endometriosis induction with the human disease.

## Conclusions

Our results suggest that the dynamic increase in the percentage of uNK cells in the secretory phase is preserved in the eutopic endometrium of women with endometriosis. Further work is indicated to assess if the observed uNK cell dynamics are perturbed in the subset of women with endometriosis who are also sub-fertile. We hypothesize that lower uNK cells associated with ectopic endometrial cells may permit the early establishment of these lesions and that NKp30 expressing uNK cells (and possibly T cells) may have a role in endometrial shedding/regeneration. However, our knowledge on the putative role of uNK cells in endometriosis is far from complete and further studies are required to explore the intricate function of these cells and explain their involvement in the pathogenic mechanisms of endometriosis.

## Additional files

Additional file 1: **Figure S1.** **A**. Graph showing correlation between CD56 and NKp30 in control human patients (*n* = 30). Spearman rank correlation *r* = 0.63, *P* = 0.0002. **B.** Graph showing correlation between CD56 and NKp30 in patients with endometriosis (n = 30). Spearman rank correlation *r* = 0.35, *P* = 0.058 (PDF 131 kb)

Additional file 2: **Table S1.** Output from bioinformatics analysis showing differential expression of genes encoding NK cell receptors and ligands in secretory phase endometrium from endometriosis patients when compared with the endometrium of control women without endometriosis ordered by specificity and overall gene score. Full gene list examined is shown in the second tab. (XLSX 26 kb)

Additional file 3: **Table S2.** The differentially expressed genes in post ovulatory eutopic endometrium of baboons 6 months after induction of endometriosis (*n* = 4) compared with the same of control animals (n = 4) published in Additional file 3: Table S2, in Afshar et al. [29] (bioset 2) was interrogated to identify 40 out of 92 genes encoding for uNK cell receptor/ligand described in 2nd tab of the Additional file 2: Table S1 (bioset 1) amongst these altered genes. *NCR3, CADM1* and *HCST* genes which were amongst the top 10 up-regulated genes in the human eutopic endometriosis (in Table 1) were amongst these, suggesting a close homology of the baboon model with the human disease (XLSX 12 kb)
