# Supplementary figures and images for: The dynamic changes in the number of uterine natural killer cells are specific to the eutopic but not to the ectopic endometrium in women and in a baboon model of endometriosis
Source: Reprod Biol Endocrinol. 2018 Jul 18;16:67. doi: 10.1186/s12958-018-0385-3 (PMC6052567; doi:10.1186/s12958-018-0385-3)

**A**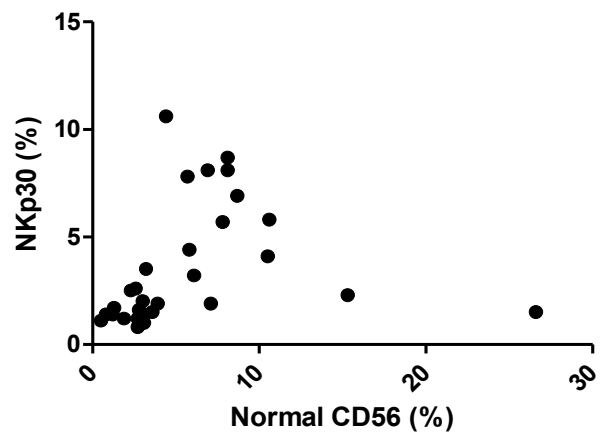**B**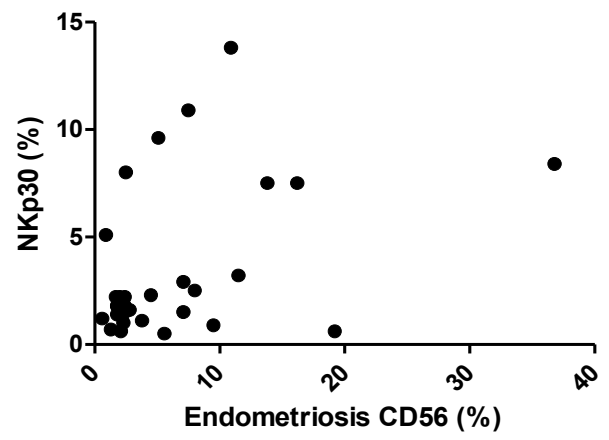

Supplement: Supplementary file 1 — Figure S1. A. Graph showing correlation between CD56 and NKp30 in control human patients (n = 30). Spearman rank correlation r = 0.63, P = 0.0002. B. Graph showing correlation between CD56 and NKp30 in patients with endometriosis (n = 30). Spearman rank correlation r = 0.35, P = 0.058 (PDF 131 kb) [file 12958_2018_385_MOESM1_ESM.pdf]
